# Validation of the Flourishing Scale in a sample of people with suboptimal levels of mental well-being

**DOI:** 10.1186/s40359-016-0116-5

**Published:** 2016-03-17

**Authors:** Marijke Schotanus-Dijkstra, Peter M. ten Klooster, Constance H. C. Drossaert, Marcel E. Pieterse, Linda Bolier, Jan A. Walburg, Ernst T. Bohlmeijer

**Affiliations:** Trimbos Institute, Department of Public Mental Health, P.O. Box 725, 3500 AS Utrecht, The Netherlands; Centre for eHealth and Well-being Research, Department of Psychology, Health and Technology, University of Twente, Enschede, The Netherlands

**Keywords:** Mental well-being, Social-psychological functioning, Eudaimonic well-being, Psychometric properties, Confirmatory factor analysis, Item response theory

## Abstract

**Background:**

There is growing interest in measuring the eudaimonic perspective of mental well-being (social and psychological well-being) alongside existing measures of the hedonic perspective of mental well-being (subjective well-being). The Flourishing Scale (FS) assesses core aspects of social-psychological functioning and is now widely used in research in practice. However, the reliability and validity of eudaimonic measures such as the FS has not yet been tested in people with low or moderate levels of well-being. This group is at risk for developing mental disorders and, therefore, an important target group for public mental health.

**Methods:**

We extensively evaluated the psychometric properties of the 8-item FS in a sample of adults with low or moderate levels of well-being in The Netherlands (*N* = 275) using confirmatory factor analysis (CFA), item response theory analysis and a multitrait matrix.

**Results:**

The unidimensional structure of the scale was confirmed with CFA and an adequate fit to the Rasch model. However, our sample showed positive skewness of the scale, but lacked measurement precision at the higher end of the social-psychological continuum. In general, the multitrait matrix demonstrated the convergent validity of the scale, with strong to weak correlations between the FS and (1) overall well-being, (2) social and psychological well-being (3) positive eudaimonic states, (4) hedonic states, (5) psychopathology and (6) personality traits. Nevertheless, relatively low correlations were found, specifically in comparison with the Mental Health Continuum-Short Form (MHC-SF).

**Conclusions:**

The FS seems a reliable and valid instrument for measuring social-psychological functioning in adults with suboptimal well-being, but its use in intervention studies and clinical practice might be debatable. Therefore, the FS seems most suitable to include in epidemiological studies alongside existing hedonic measures to more fully capture mental well-being. Future research should examine the temporal stability of the FS and the consequences of the positive skewness and limited external validity of the scale found in the current study.

## Background

Mental well-being is an important multifaceted construct with an extensive and long-standing history in the scientific literature. Mental well-being captures both the hedonic and eudaimonic perspectives on well-being. Whereas the hedonic perspective refers to the affective or ‘feeling good’ dimension of well-being (i.e. happiness, life-satisfaction and positive affect) [1] the eudaimonic perspective refers to the psychological functioning or ‘living well’ dimension of well-being (e.g. social contribution, positive relationships with others and personal growth) [2, 3]. Traditionally, most (socioeconomic) research has been conducted on the hedonic perspective with the use of single-item or brief subjective well-being measures such as the Satisfaction with Life Scale [4], the Subjective Happiness Scale [5] and the Positive and Negative Affectivity Scale [6]. While these measures are firmly rooted in research and practice, the availability and use of measures to capture the social and psychological well-being dimensions lags behind.

A few decades ago, researchers started to unravel the core aspects of the eudaimonic perspective. Ryff [3] defined six core dimensions of psychological well-being (self-acceptance, purpose in life, environmental mastery, positive relationships, personal growth and autonomy), based on an extensive review of humanistic, existential and developmental theories. Keyes [2] identified five core dimensions of social well-being (social acceptance, social actualization, social contribution, social coherence and social integration) originating from sociological and social psychological theories. These social and psychological core aspects of well-being are united in the comprehensive Mental Health Continuum-Short Form (MHC-SF) which also measures hedonic (subjective) well-being [7, 8]. There are a few other comprehensive generic well-being instruments available, such as the WHO Five Well-being Index [9] and the Warwick-Edinburgh Mental Well-being Scale [10]. However, in order to complement existing measures of the hedonic perspective, for example in epidemiological and socioeconomic research, there is also a need for instruments assessing only the core dimensions of the eudaimonic perspective. Therefore, Diener et al. [11] have recently developed the brief and comprehensive Flourishing Scale (FS) based on humanistic and eudaimonic well-being theories.

Today, the FS is widely used in well-being intervention studies and clinical practice, probably due to its briefness, simplicity and comprehensiveness. The FS has already been translated into 17 languages and measures the core aspects of social-psychological functioning, namely purpose and meaning, supportive relationships, engagement, contribution to the well-being of others, competence, self-acceptance, optimism, and being respected. The growing popularity of the FS might also be a consequence of its attractive name, suggesting that the scale measures ‘flourishing’. However, most researchers have defined flourishing as a state where *high levels of subjective* well-being and *high levels of social-psychological* well-being are achieved [12–14]. As such, the scale’s name may be somewhat confusing because it only measures social-psychological well-being and lacks a clear cut-off for having high levels of social-psychological well-being. Regarding the development of the scale, its first version was labeled the Psychological Flourishing Scale and contained 12 items [15]. The revised and final version of the scale has eight items and was called the Psychological Well-being scale [16]. Since this name was so similar to Ryff’s Scales of Psychological Well-being [3], the authors re-named their scale to the FS [11].

Acceptable psychometric properties of the FS have been found in student samples [11, 17, 18], a full-time employee sample [19], a community sample [20] and in a national representative population sample [21]. All these studies found a single factor structure using exploratory or confirmatory factor analysis (EFA and CFA), and adequate to excellent reliability with Cronbach’s alpha values ranging from .78 to .95. Most previous validation studies also supported the convergent validity of the FS. For example, moderate to strong positive correlations were found for overall psychological well-being (i.e. Ryff’s Scales of Psychological Well-being and the Basic Needs Satisfaction Scale) and moderate to strong negative correlations were found for depression, anxiety and stress [11, 18, 21]. Yet, the convergent validity of the FS has mostly been supported by measures of subjective well-being (i.e. happiness, life-satisfaction and positive emotions) only [11, 18, 19, 21]. Since the FS measures core aspects of optimal social-psychological functioning, more information is needed about how each of these core aspects (such as competence, self-compassion and positive relationships) are related to the FS. In addition, the relationship between eudaimonic well-being and personality traits has hardly been investigated, although there are some indications that weak correlations can be expected. For example, Lamers and colleagues [22] found weak positive correlations between subjective, psychological and social well-being on the one hand and emotional stability (opposite of neuroticism), extraversion and conscientiousness on the other.

The current study adds to the psychometric validation of the FS in several ways. First, we evaluated the internal and external construct validity of the FS in a sample of adults with low or moderate levels of well-being which seems an important target group for public mental health and well-being intervention studies because low to moderate well-being increases the risk of developing mental illness [23–26]. Second, we used item response theory (IRT) analyses to further demonstrate the unidimensionality of the FS and explore its local reliability (measurement precision) along the underlying continuum. Third, we further unraveled the convergent validity of the FS using a multitrait matrix. With respect to convergent validity, we expected to find the following gradual pattern of stronger to weaker relationships with (1) overall well-being, (2) social and psychological well-being, (3) positive eudaimonic states (i.e. the use of strengths/competence, optimism, self-compassion, resilience and positive relationships), (4) hedonic states (i.e. emotional well-being, positive and negative emotions), (5) psychopathology (i.e. anxiety, depression) and (6) personality traits (i.e. extraversion, neuroticism, conscientiousness).

## Method

### Participants

We used data from the baseline measurement of a randomized controlled trial in The Netherlands that evaluated the efficacy of a multicomponent positive psychology intervention [27]. Participants with low or moderate levels of well-being were recruited in January 2014 by advertisements in national newspapers and in an online newsletter of a popular psychology magazine. In total, 275 participants were included, gave informed consent and completed the online survey at baseline. Mean age was 47.8 years (SD = 10.9) with a range from 20 to 67 years. Participants were mainly female (85.8 %), higher educated (74.6 %) and in paid employment (67.6 %) (Table 1). While we had excluded individuals with flourishing mental health (i.e. high levels of both hedonic and eudaimonic well-being) two weeks prior to baseline, at baseline there were 21 (7.6 %) respondents who met the classification criteria for flourishing as measured with the MHC-SF [8].

**Table 1:** Demographic characteristics of the study sample (*N* = 275)

Characteristic	Mean ± SD or number (%)
Age, years	47.78 ± 10.88
Gender, female	236 (85.8 %)
Education	
Low	10 (3.7 %)
Middle	59 (21.7 %)
High	203 (74.6 %)
Marital status	
Married / Registered	118 (42.9 %)
Divorced / Widow	70 (25.5 %)
Never been married	87 (31.6 %)
Living situation	
With partner and children	78 (28.4 %)
With partner without children	76 (27.6 %)
Alone	76 (27.6 %)
With others	45 (16.4 %)
Daily activities	
Paid employment	186 (67.6 %)
Unemployed / Unable to work	58 (21.1 %)
Other	31 (11.2 %)

*SD* standard deviation

### Measures

The FS [11] consists of eight items, each measuring a core aspect of optimal social-psychological functioning on a 7-point Likert scale that ranges from 1 (Strongly disagree) to 7 (Strongly agree). For this study, the original English version of the FS was independently translated into Dutch by two bilingual native Dutch speakers (authors PMK and ETB). Both translations were compared and inter-translator differences were carefully discussed before consensus was reached. The Dutch version was used in the current study. All other measures were also administered in Dutch.

The MHC-SF [8, 28] consists of 14 items which are divided into the three subscales ‘emotional well-being’ (three items), ‘social well-being’ (five items), and ‘psychological well-being’ (six items). In addition, the scores on all 14 items can be averaged into a total well-being score. Items are answered on a 6-point scale ranging from 0 (never) to 5 (almost always or always).

The Strength Use Scale (SUS) measures the level of competence in different settings with 14 items. Each item is answered on a 6-point scale that ranges from 1 (Strongly disagree) to 7 (Strongly agree) [29].

The Life Orientation Test-Revised (LOT-R) consists of 10 items that assess dispositional optimism versus pessimism [30]. Four filler items were excluded from this analysis and of the remaining six, three items measure optimism and three measure pessimism. The items are answered on a 5-point scale with a range from 0 (Strongly disagree) to 4 (Strongly agree). A total score was obtained for a more optimistic expectation about the future.

The Self-Compassion Scale-Short Form (SCS-SF) is a 12-item measurement used to assess the level of self-compassion on a 7-point scale that ranges from 1 (Rarely or never) to 7 (Almost always) [31].

The Brief Resilience Scale (BRS) is a 6-item inventory that assesses the ability to bounce back and to cope with stress or negative life-events [32]. Answers are given on a 5-point scale that ranges from 1 (Strongly disagree) to 5 (Strongly agree).

Ryff’s Subscale of Positive Relationships (SPR) is a subscale of Ryff’s Scales of Psychological Well-being [3]. The SPR has 9 items and a 6-point answer scale that ranges from 1 (Strongly disagree) to 6 (Strongly agree) [33].

Positive and negative emotions were assessed with the modified Differential Emotions Scale (mDES), which measures the frequency of eight groups of positive emotions and feelings and eight groups of negative emotions and feelings, with answer categories on a 7-point scale that ranges from 1 (Not at all) to 7 (Very intense) [34].

Depression and anxiety symptoms were measured with the Hospital Anxiety and Depression Scale (HADS), an inventory with two subscales that assess the frequency of anxiety symptoms (seven items) and depression symptoms (seven items) [35]. Answer categories differ for each item, but all items are answered on a 4-point scale (0–3). This questionnaire was administered at screening, around two weeks before the baseline measurement. Participants with a score above 10 on one or both subscales were excluded from the randomized controlled trial and, therefore, also from the current study.

In accordance with the national representative Netherlands Mental Health Survey and Incidence Study-2 [36], we measured the personality traits extraversion and neuroticism with the Eysenck Personality Questionnaire-Revised Short Scale (EPQ-RS) [37] and conscientiousness with the 12-item NEO Five Factor Inventory (NEO-FFI) [38]. Extraversion and neuroticism were each measured with 12 items answered with yes (1) or no (0). The NEO-FFI has a 5-point scale that ranges from 1 (Strongly disagree) to 5 (Strongly agree).

### Statistical analyses

Descriptive and distributional statistics of the FS total scores were determined by the identification of possible skewness, kurtosis, and floor and ceiling effects. Skewness and kurtosis values between -1 and +1 were considered indicative of normality, and floor and ceiling effects were considered present when more than 15 % of the participants scored the lowest (8) or highest possible score (56) [39]. There were no missing values on any of the measures used in this study.

Given the ordinal nature of the items, the unidimensionality of the FS was examined using robust maximum likelihood CFA with Satorra-Bentler (SB) scaled statistics. With the use of LISREL version 8.80 (Scientific Software International, Lincolnwood, IL), a one-factor model was fit to the data. We did not allow error covariances between items (i.e. shared item variance) because each item corresponds to one core aspect of social-psychological functioning which are theoretically distinct. Indicators of a good model fit were a non-normed fit index (NNFI) and comparative fit index (CFI) ≥ .95, standardized root mean square residual (SRMS) ≤ .08, and root mean square error of approximation (RMSEA) ≤ .06 [40, 41]. The internal consistency of the FS was examined with Cronbach’s alpha, with a value ≥ .70 considered adequate for group-level analyses [42].

To further determine the internal construct validity of the FS, we performed Rasch partial credit model analyses in Winsteps version 3.65 (Winsteps, Chicago, IL). The Rasch partial credit model is an extension of the original dichotomous Rasch model specifically designed for ordinal scales. Fit to the Rasch model provides further evidence of unidimensionality, but also allows the investigation of a scale’s local measurement precision. Regarding the former, indicators of acceptable item fit were mean square infit (information-weighted fit statistic) and outfit (outlier sensitive fit statistic) values between .70 and 1.30 [43]. The infit statistic is sensitive to outliers on those items that are close to the abilities of a person, and the outfit statistic is sensitive to outliers on all items independent from the person’s level of well-being [44]. Also, a test information curve was obtained for examining the local measurement precision of the scale along the latent social-psychological well-being continuum. Overall reliability was examined with the person reliability measure, which is the Rasch-based version of Cronbach’s alpha. Rasch person reliability is the proportion of observed variance that is free from measurement error. In practice, values around .80 are considered acceptable [45].

The external construct validity of the FS was examined with a wide variety of measures as detailed above. Pearson correlation coefficients were used to evaluate the convergent validity as a gradual pattern of decreasing correlations with an expected strongest relation between FS and MHC-SF and an expected weakest relation between FS and personality traits. Bivariate correlations were obtained using SPSS version 21.0 (IBM, Chicago, IL).

## Results

### Descriptive analysis

**Fig. 1:**
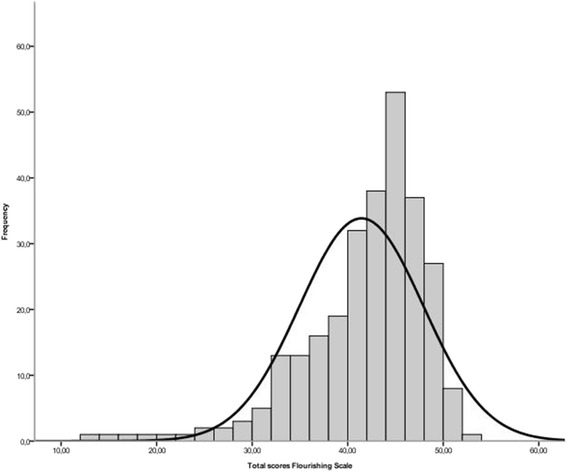
Distribution of total scores of the Flourishing Scale

The mean total score on the FS was 41.4 [Standard deviation (SD) = 6.5] with a range from 13 to 53. Mean scores on the individual items ranged from 4.7 to 5.5, on a scale of 1 to 7. Figure 1 shows the distribution of the total scores on the FS, which were skewed towards higher scores on optimal social-psychological functioning (Kolmogorov-Smirnov, *p* < .001), with a skewness value of −1.46 and a kurtosis value of 2.99. There were no floor or ceiling effects since no participants scored either 8 or 56. These descriptive and distribution statistics suggest that the majority of our sample perceived themselves positively on the main aspects of social-psychological functioning.

### Internal construct validity

**Fig. 2:**
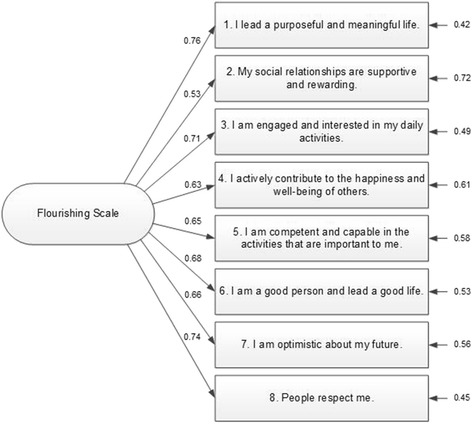
Standardized factor loadings and residuals for the eight items of the Flourishing Scale

Results of the CFA revealed good fit indices for a one-factor model, supporting the unidimensional structure of the FS. All indices were within the recommended range for good fit: SB χ^2^ (20) = 39.59, NNFI = .98, CFI = .99, SRMR = .05, RMSEA (90 % CI) = .06 (.03–.09). Figure 2 shows the standardized factor loadings and the item residuals. Factor loadings ranged between .53 for item 2 and .76 for item 1. Additionally, the FS showed good internal consistency with a Cronbach alpha coefficient of .86.

**Fig. 3:**
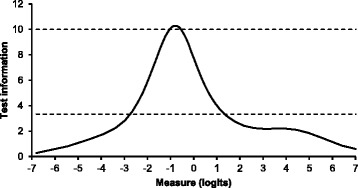
Test information curve of the Flourishing Scale (FS) in relation to the Rasch score. Higher positive logit scores indicate higher social-psychological functioning. Test information values of 3.33 and 10 (dashed lines) correspond to a reliability of .70 and .90, respectively. Logit values of −7, −3, 0, 1, and 7 correspond to approximate total sum scores on the FS of 11, 16, 38, 43, and 55 respectively

The unidimensionality of the scale was further confirmed by an adequate fit to the Rasch model. Most items performed within the range of good fit (0.7–1.3), except for minor misfit of the infit and outfit values for item 2 and the outfit value for item 5 (Table 2). The item difficulty in logits shows that item 1 was the most difficult to endorse (1.01 logits) and item 8 was the easiest to endorse (−.65). The Rasch person reliability was .79, indicating adequate reliability for group-level comparisons. However, the test information curve (Fig. 3) showed that the scale had adequate measurement precision across a rather limited range of the continuum with a clear peak at relatively lower to moderate levels of well-being (*r* > .70). Logits of this peak were between −2.7 and 1.2 and correspond to approximate total sum scores on the FS between 16 and 43. In other words, the level of optimal well-being in our sample was measured most accurately in individuals with average or below average levels of social-psychological functioning. The assessment in individuals with high levels of social-psychological functioning was less accurate.

**Table 2:** Rasch item parameters (partial credit model) and fit statistics of the Flourishing Scale

	Item difficulty in logits (SE)	Infit MNSQ	Outfit MNSQ
1. I lead a purposeful and meaningful life	1.01 (0.07)	0.81	0.86
2. My social relationships are supportive and rewarding	−0.24 (0.07)	1.35	1.34
3. I am engaged and interested in my daily activities	0.08 (0.08)	0.89	0.87
4. I actively contribute to the happiness and well-being of others	−0.30 (0.08)	0.98	0.95
5. I am competent and capable in the activities that are important to me	0.21 (0.08)	1.08	1.47
6. I am a good person and live a good life	−0.47 (0.08)	0.93	0.88
7. I am optimistic about my future	0.35 (0.07)	1.05	1.01
8. People respect me	−0.65 (0.09)	0.84	0.78

Higher positive logit scores indicate more difficult items. *SE* standard error, *MNSQ* mean square

### External construct validity

Table 3 shows that the FS correlated most strongly with the MHC-SF (*r* = .58), followed by its subscales for social and psychological well-being (*r* = .50–.58). The FS also showed a strong correlation with use of strengths (*r* = .55). Moderate to strong correlations were found for most other positive eudaimonic states (*r* = .35 to .46) and for the relation between the FS and emotional well-being (*r* = .40). We found weak to moderate correlations for other indicators of hedonic states (*r* = .15 and −.19), for psychopathology (*r* = −.17 and −.34) and for personality traits (*r* = .23 to .32). Contrary to our expectations, the weakest correlations with the FS were found for positive emotions, negative emotions, anxiety symptoms and resilience (*r* = .15 to −.19), and not for personality traits. All correlations with the FS were statistically significant (*p* < .01) and in the expected direction.

**Table 3:** Multitrait matrix of the Flourishing Scale and other measures

	FS	MHC-SF	SWB	PWB	SUS	SCS-SF	LOT-R	SPR	BRS	EWB	mDES Pos	mDES Neg	HADS-A	HADS-D	Extr	Neur
FS	(.86)															
1. Overall well-being																
MHC-SF	.58***	(.88)														
2. Social and psychological well-being																
SWB	.50***	.88***	(.70)													
PWB	.58***	.92***	.68***	(.79)												
3. Positive eudaimonic states																
SUS	.55***	.58***	.47***	.60***	(.95)											
SCS-SF	.35***	.58***	.48***	.54***	.42***	(.85)										
LOT-R	.45***	.50***	.42***	.46***	.39***	.46***	(.74)									
SPR	.46***	.48***	.44***	.47***	.29***	.30***	.32***	(.82)								
BRS	.16**	.31***	.18**	.33***	.33***	.46***	.36***	.11	(.83)							
4. Hedonic states																
EWB	.40***	.80***	.56***	.66***	.39***	.48***	.43***	.32***	.31***	(.80)						
mDES Pos	.15*	.22***	.13*	.25***	.19**	.08	.15*	.08	.10	.20**	(.56)					
mDES Neg	−.19**	−.23***	−.19**	−.21**	−.13*	−.20**	−.15*	−.24***	−.13*	−.23***	.27***	(.72)				
5. Psychopathology																
HADS-A	−.17**	−.27***	−.28***	−.22***	−.13*	−.36***	−.24***	−.12*	−.15**	−.22***	−.05	.27***	(.76)			
HADS-D	−.34***	−.49***	−.36***	−.44***	−.27***	−.31***	−.30***	−.28***	−.22***	−.52***	−.27***	.22***	.34***	(.76)		
6. Personality traits																
Extraversion	.23***	.29***	.25***	.28***	.36***	.18**	.23***	.34***	.24***	.20**	.09	−.11	.05	−.13*	(.84)	
Neuroticism	−.24***	−.40***	−.31***	−.37***	−.24***	−.55***	−.41***	−.31***	−.53***	−.36***	−.06	.21***	.39***	.24***	−.15*	(.78)
Conscient-iousness	.32***	.29***	.20**	.33***	.37***	.17**	.18**	.17**	.24***	.20**	.10	−.10	−.07	−.26***	.09	−.14*

*BRS* Brief Resilience Scale, *EWB* Emotional well-being subscale of the MHC-SF, *Extr* Extraversion, *FS* Flourishing Scale, *HADS-A* Hospital Anxiety and Depression Scale-Anxiety Subscale, *HADS-D* Hospital Anxiety and Depression Scale-Depression Subscale, *LOT-R* Life Orientation Test-Revised (optimism), *mDES Neg* modified Differential Emotions Scale, negative emotions, *mDES Pos* modified Differential Emotions Scale, positive emotions, *MHC-SF* Mental Health Continuum-Short Form, *Neur* Neuroticism, *PWB* Psychological well-being subscale of the *MHC-SF*, *SCS-SF* Self-compassion Scale-Short Form, *SPR* Ryff’s Subscale of Positive Relationships, *SUS* Strengths Use Scale, *SWB* Social well-being subscale of the MHC-SF

Cronbach’s alphas are in parentheses. Coefficients ≥ .50 are in bold

* *p* < .05, ** *p* < .01, *** *p* < .001

## Discussion

This is the first study to evaluate the internal and external construct validity of the Flourishing Scale in a sample of 275 adults with suboptimal well-being. Robust CFA and item response theory analysis supported prior findings for the unidimensionality of the scale and demonstrated satisfactory item fit. However, the Rasch results also showed that social-psychological functioning was most adequately measured across a rather limited range of its continuum. The convergent validity of the FS was partially supported by our data.

### Internal validity

The Rasch analysis demonstrated adequate overall reliability and good item fit for most items. However, there was some misfit for item 2 (i.e. ‘My social relationships are supportive and rewarding’) and item 5 (i.e. ‘I am competent and capable in the activities that are important to me’). Misfit values slightly exceeded the boundary of 1.30, suggesting that these items showed more observed variance than was expected in the model. Replications in other samples are needed to determine whether revision of the items is necessary.

An intriguing result concerns the positive skewness of the FS. Despite the fact that we excluded individuals with high levels of well-being (i.e. flourishing mental health) two weeks before the baseline measurement—as measured with the MHC-SF—the total scores on the FS were rather skewed towards higher social-psychological functioning in agreement with prior validation studies. For example, our mean score of 41.4 (SD = 6.5) is only slightly lower than the mean scores found in a general population sample (mean = 43.8, SD = 8.4) [21], an employee sample (mean = 42.9, SD = 6.1) [19] and different student samples (most means were between 44.5 and 46.7 [11, 17, 19], except for one study that found a mean score of 36.6 [18]). Thus, while the MHC-SF and FS both predominantly intend to measure the eudaimonic perspective of well-being, our findings could indicate that both instruments actually measure different aspects of optimal social-psychological functioning. For example, the FS contains items about competence, engagement and optimism while these eudaimonic aspects are not specifically questioned in the MHC-SF [14]. More research into consequences of the positive skewness of the FS is needed, for instance, by validating the FS in clinical samples where a more normally-distributed level of social-psychological well-being could be expected.

Moreover, our research also demonstrated that social-psychological functioning was most reliably measured between scores of 16 and 43, scores that correspond to the ‘very low’ and ‘low’ population norm classifications of Diener [46]. Thus, participants in a variety of samples (including the present sample) tend to score high on the FS, but measurement precision in the present sample showed that high social-psychological functioning was less adequately measured. Therefore, our results suggest that the FS may benefit from more differentiation in the difficulty of the items by including items that are indicative for higher levels of well-being or items that can better discriminate between the moderate and high end of the social-psychological continuum. Measurement precision across a broader range of the continuum is especially important when researchers want to examine individual changes in well-being scores and the transition from low or moderate well-being to high well-being, which is often the main aim in well-being intervention studies. Overall, the operationalization of eudaimonic well-being and its core aspects warrant further investigation, as well as research about adequate cut-off values for ‘high eudaimonic well-being’. In this regard, it should be recognized that little information is available about the theoretical foundation of the FS [11], especially concerning a solid overarching theory and the rationale for including some eudaimonic concepts whilst ignoring others.

### External validity

Regarding convergent validity, our results largely confirmed the hypothesized gradual pattern of descending correlations between the FS and the MHC-SF, positive eudaimonic states, hedonic states, psychopathology, and personality traits respectively. While we found a strong relationship between the FS and MHC-SF, its correlation of *r* = .58 was considerably lower than a priori could be expected. Noteworthy, the MHC-SF showed a similar gradual pattern of correlations from closely to more distant related measures, but with consistently higher correlations compared to the FS. Despite the fact that the MHC-SF showed moderate to strong correlations with depression and neuroticism, these findings suggest that the MHC-SF may have superior convergent validity compared to the FS.

Strikingly, we found lower than expected correlations between the FS and most positive eudaimonic states, such as self-compassion and optimism. An explanation might be that the items of the FS are too broadly phrased which may diffuse their relation with their underlying individual constructs. Another explanation might be that our sample was too homogenous by excluding people with flourishing mental health. However, prior studies also found predominantly moderate to strong correlations with eudaimonic well-being measures of which the highest correlations were only around .70 for some subscales of Ryff’s Scales of Psychological Well-being and the ‘competency’ subscale of the Basic Needs Satisfaction Scale [11].

Furthermore, while the hedonic perspective is not represented in the eight items of the FS, the relationship between the FS and emotional well-being was not much lower than for the observed correlations between the FS and measures of positive eudaimonic states. This result corroborates with the view that hedonic and eudaimonic well-being are distinct but overlapping perspectives of well-being [47]. However, we found lower correlations compared to prior FS validation studies which found moderate or even strong correlations between the FS and measures of the hedonic perspective [11, 18, 19, 21], raising again the question how eudaimonic well-being should be operationalized [48]. In sum, our findings may point to limited external validity of the FS, at least in comparison with the MHC-SF.

### Limitations

Our study was limited by the representativeness of the sample. Participants were self-selected adults with low or moderate levels of well-being (without elevated levels of clinical symptomatology) who were motivated to improve their well-being with a positive psychology intervention. Also, female and highly-educated participants were overrepresented. Another limitation of the study was the inability to examine the temporal stability and responsiveness of the FS in our study. The test-retest reliability of the scale has only been examined by Diener and colleagues [8], who used a student sample and a time interval of one month. For the use of the FS in longitudinal and intervention studies, it is essential that future studies establish the stability of the FS and its sensitivity to change. Finally, due to the low Cronbach’s alpha of .56 for positive emotions in our sample, its correlational results should be interpreted with some caution.

## Conclusion

Researchers, practitioners and governments are increasingly interested in the concept of mental well-being and flourishing, but the majority of epidemiological studies have only included brief subjective well-being measures (typically containing one to five items) alongside economic, social and health indicators [38]. Therefore, it seems important to include the FS as a complementary measure to more fully capture mental well-being in the general population. From a public mental health and societal perspective, it is also important to improve social-psychological functioning because flourishing protects against the development of mental disorders later in life [39–42]. The current study indicates that the FS might be most appropriate for use in epidemiological studies alongside an existing hedonic measure, but its use in well-being intervention studies and clinical practice might be debatable. In particular, we found positive skewness of the FS in a sample of people with suboptimal well-being, the FS lacked measurement precision at higher levels of social-psychological functioning and demonstrated relatively low correlations with overall well-being and positive eudaimonic states. In sum, the Dutch version of the FS appears to be a reliable tool for measuring the core aspects of the eudaimonic perspective in adults with low or moderate levels of well-being, but researchers and practitioners should be aware of its possible limitations as a standalone measure of flourishing.

### Ethics approval and consent

This study was approved by the Ethics Committee of the University of Twente (number 13212). All participants gave online informed consent.

### Availability of data and materials

The data used in this study are available upon request from the corresponding author.

## Abbreviations

BRS: Brief Resilience Scale
CFA: confirmatory factor analysis
CFI: comparative fit index
EFA: exploratory factor analysis
EPQ-RSS: Eysenck Personality Questionnaire-Revised Short Scale
EWB: emotional well-being subscale
Extr: extraversion
FS: Flourishing Scale
HADS: Hospital Anxiety and Depression Scale
IRT: item response theory
LOT-R: Life Orientation Test-Revised
m-DES: modified Differential Emotions Scale
MHC-SF: Mental Health Continuum-Short Form
MNSQ: mean square
NEO-FFI: NEO Five Factor Inventory
Neur: neuroticism
NNFI: non-normed fit index
PWB: psychological well-being subscale
RMSEA: root mean square error of approximation
SB: Satorra-Bentler
SCS-SF: Self-Compassion Scale-Short Form
SD: standard deviation
SE: standard error
SPR: Subscale of Positive Relationships
SRMS: standardized root mean square residual
SUS: Strength Use Scale
SWB: social well-being subscale
